# Relationship between low birth weight and infant mortality: evidence from National Family Health Survey 2019-21, India

**DOI:** 10.1186/s13690-023-01037-y

**Published:** 2023-02-21

**Authors:** Arup Jana, Unnati Rani Saha, R. S. Reshmi, T. Muhammad

**Affiliations:** 1 International Institute for Population Sciences, Deonar, Mumbai, 400088 India; 2 Department of Public Health, Erasmus MC, University Medical Center Rotterdam, Rotterdam, The Netherlands

**Keywords:** LBW, Infant mortality, BMI, Institutional delivery, Preterm birth, Antenatal care

## Abstract

**Background:**

Low birth weight (LBW) predisposes physical and mental growth failure and premature death among infants. Studies report that LBW predominately explains infant mortality. However, existing studies rarely demonstrate the phenomenon of both observed and unobserved factors, which may influence the likelihood of birth and mortality outcomes simultaneously. In this study, we identified the spatial clustering of the prevalence of LBW along with its determinants. Further, the relationship between of LBW and infant mortality, considering the unobserved factors, has been explored in the study.

**Methods:**

Data for this study have been extracted from the National Family Health Survey (NFHS) round 5, 2019-21. We used the directed acyclic graph model to identify the potential predictors of LBW and infant mortality. Moran’s I statistics have been used to identify the high-risk areas of LBW. We applied conditional mixed process modelling in Stata software to account for the simultaneous nature of occurrences of the outcomes. The final model has been performed after imputing the missing data of LBW.

**Results:**

Overall, in India, 53% of the mothers reported their babies’ birth weight by seeing health card, 36% reported by recall, and about 10% of the LBW information was observed as missing. The state/union territory of Punjab and Delhi were observed to have the highest levels of LBW (about 22%) which is much higher than the national level (18%). The effect of LBW was more than four times larger compared to the effect in the analysis which does not account for the simultaneous occurrence of LBW and infant mortality (marginal effect; from 12 to 53%). Also, in a separate analysis, the imputation technique has been used to address the missing data. Covariates’ effects showed that female children, higher order births, births that occur in Muslim and non-poor families and literate mothers were negatively associated with infant mortality. However, a significant difference was observed in the impact of LBW before and after imputing the missing values.

**Conclusions:**

The current findings showed the significant association of LBW with infant deaths, highlighting the importance of prioritising policies that help improve the birth weight of new-born children that may significantly reduce the infant mortality in India.

## Introduction

India plays a crucial role in ending premature deaths as the nation carries the highest number of child mortality globally [1]. Infant mortality rate (IMR) declined from 81 to 35 per 1000 live births between 1990 and 2016, with a 1.3% reduction rate [2]. The sustainable development goals (SDG) envisage to end preventable deaths of new-borns and children under 5 years of age, with all countries aiming to reduce neonatal mortality to at least as low as 12 per 1000 live births and under-5 mortality to as low as 25 per 1000 live births by 2030 [2]. Unfortunately, the IMR in India is much higher than in the neighbouring countries, i.e. Nepal, Bangladesh, and Sri Lanka [1]. A study stated that due to the regional variations, half of the districts in India are unlikely to achieve the SDG target of child mortality by 2030 [3].

In India, approximately 83% of neonatal deaths occur due to complications from low birth weight (LBW) [4]. Pprevious studies have reported that infants born with a weight less than 2.5 kg face a higher risk of malnutrition and childhood morbidities such as diarrhoea and pneumonia [5, 6], which are the leading causes of neonatal and child mortality [7] and remain as an alarming concern among policymakers [8]. Furthermore, those who survive with LBW are more likely to experience problems related to cognitive capacity, attainment of schooling, degenerative disorder and growth faltering, which affect their income and productivity [5, 9, 10]. Child malnutrition, which is one of the results of LBW, alone accounts for half of the global child deaths [11]. Therefore, understanding the determinants of LBW is necessary to reduce mortality and improve the development indicators for future generations, especially in the limited resource countries such as India.

The etiology of LBW is not clearly understood. A study in South Africa demonstrated that the lack of antenatal care, hypertensive disorder during pregnancy, and previous cesarean delivery are associated with LBW [12]. Also, mother’s weight and height are significantly associated with LBW [13]. Additionally, mother’s education, wealth status, and birth order of the infant are the critical contributors to LBW [14]. Further, maternal nutritional status plays a significant role in the growth of the fetus, which is one of the important determinants of LBW [15]. Previous studies stated that household sanitation facilities, drinking water, and cooking fuel are the significant contributors to infant deaths [16, 17]. Therefore, the rationale for the present study is to understand the factors explaining LBW and its effects on their survival during their infancy. Also, interest lies in identifying the spatial clustering of LBW. We hypothesized that there are some common maternal and child health care factors, which can be useful to reduce the burden of both LBW and infant mortality. Thus, it would be helpful for the policymakers to relook into the strategies to achieve the third goal of SDGs, that is, ensuring good health and wellbeing for all at all ages in limited resource countries.

### Conceptual framework

**Fig. 1:**
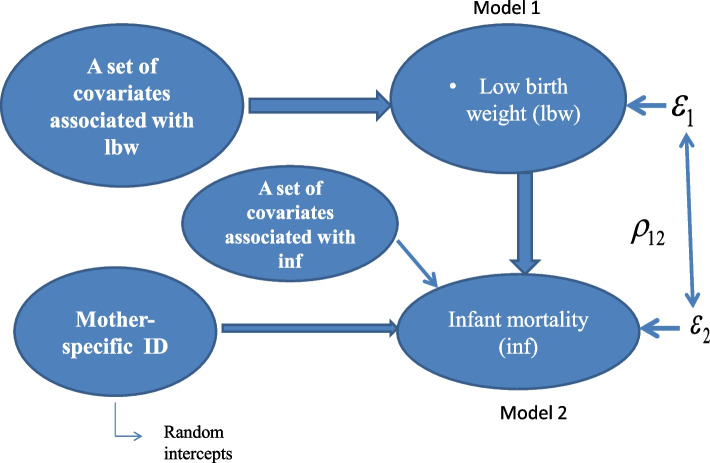
The conceptual framework of the study

The study investigates the association between LBW and infant mortality. In the conceptual model, Fig. 1 displays the mechanism that is investigated in the present study. In social science research it is sometimes unable to observe the relevance of an occurrence. For example, in this study, the unobserved characteristics refer to maternal biological factors including factors that are not directly observed in the data. Therefore, it is difficult to explain the correlated error term as they can be correlated in many ways. However, without controlling for this, it may bias the covariates’ effect in the model and conclusions on risk factors. Therefore, a child born with LBW may depend on unobserved maternal characteristics (among explanatory variables). Similarly, infant mortality may depend on (among explanatory variables) unobserved maternal characteristics. For example, some mothers are more prone to give birth with LBW, leading to infant death. This is a common phenomenon for observations for the same unit that are influenced by the same (shared) unit-specific time unobserved invariant heterogeneity. Ignoring such indignity (confounding) may lead to biased inference of the impact of LBW on infant death. Our model accounted for cross equation correlation where the idiosyncratic error term (ε_1_, ε_2_) of each equation allowed to be correlated. Failing to include potential observed covariates in the model might lead to an unobserved heterogeneity (unobserved variability) in the response variable. For instance, potential covariates of LBW such as ‘maternal smoking during pregnancy, hypertensive disorder during pregnancy, which we cannot enter in our LBW model because of unavailability; thus, it remains unobserved and included in the idiosyncratic error term. Similarly, these factors may influence the mother’s behavior, a shared factor that influences the death of her child in an unobserved way.

## Methodology

### Data

The National Family Health Survey (NFHS), round 5 is a nationally representative survey that collected information on a wide range of socio-demographic and health indicators, conducted from June 2019 to April 2021 across 36 states and union territories. Census enumeration areas have been considered the primary sampling unit at the first stage of the sampling frame. In the second stage, households have been randomly selected from the sampling frame’s primary sampling unit, ‘cluster’. The details of the sampling technique are published elsewhere [18]. In the nationally representative study, data have been collected by using computer-assisted personal interviewing (CAPI). The response rate was 97%. The NFHS-5 survey collected information of 232,920 children during the survey period. The survey asked about the birth weight using the following questions: ‘Was weighed at birth?’ if yes, ‘How much did weight’ [18]. Mothers reported in two ways; the first is by remembering the baby’s weight, and the second is by using any card. The data revealed that 7924 children died before completing their first year of birth.

The NHFS has been conducted under the authority of the International Institute for Population Sciences (IIPS), under the Ministry of Health and Family Welfare. Ethical approval for the survey has been taken from the research ethics committee of the Ministry of Health and Family Welfare. Further, informed consent has been taken from each respondent before enrolling in the survey.

### Dependent variables

Based on the question on the information of birth weight collected by the NFHS survey, LBW was defined as per the definition of the World Health Organization (WHO); with weight at birth less than 2500 grams. We created a dichotomous variable of birth weight based on the definition: ‘1’ for LBW and ‘0’ for no LBW.

Infant mortality is defined as a child’s death before completing the first year of life [19]. The survey collected information on the child’s survival status; whether the child was ‘died’ or ‘alive’. If the child is not surviving, they asked about the child’s age at death. The dependent variable is categorized into a dichotomous variable; ‘0’ for alive children and ‘1’ for infant deaths.

### Directed Acyclic Graph (DAG) model to select the covariates

**Fig. 2:**
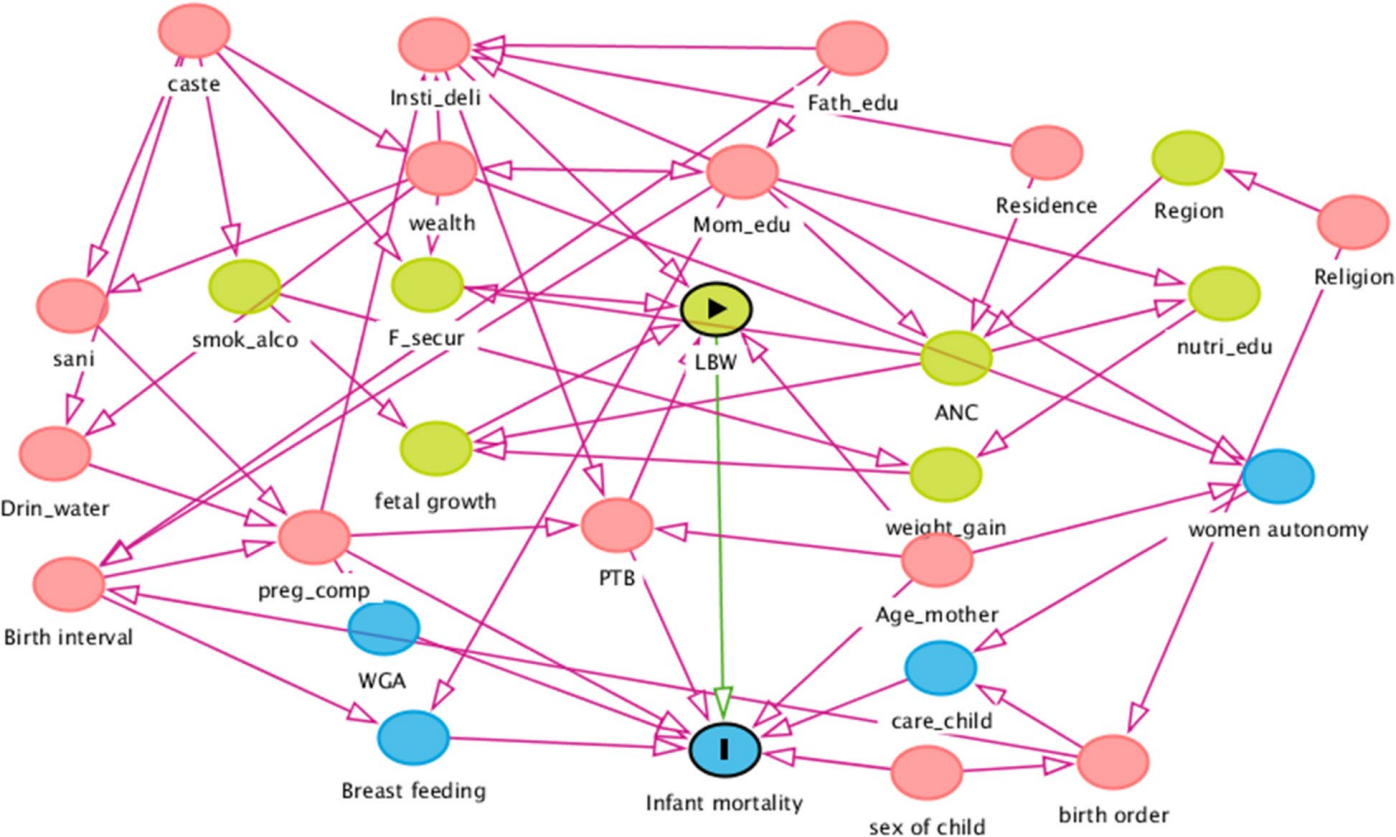
Direct Acyclic Graph (DAG) used for selecting control variables in the study

The DAG model was used to establish a causal inference in epidemiology to illustrate how associations translate into causal relationships. Epidemiologists mostly use DAG model to identify the mediators and moderators of the casual relationship between predictor and outcome variables that is also useful to identify the actual covariates. In the DAG model (Fig. 2), the blue circle with a bar indicates the outcome variable of the study. The yellow circle with a triangle represents the main exposure variable. A blue and yellow circle indicates the ancestors of outcome and exposure variables, respectively. The red circles represent the ancestor of exposure and outcome variable. The green and red lines indicate the causal and open pathways (where confounding might occur), respectively. The confounding pathways can be avoided by adjusting the observed and unobserved variables on the pathway.

Insti_deli = Institutional delivery, Fath_edu = Father’s education, Mom_edu = Mother’s Education, wealth = Wealth status of the household, smok_alco = Consumption of alcohol or smoking, F_secur = Food security, Sani = Sanitation facility of the household, Drin_water = Sources of drinking water, LBW = Low birth weight, preg_comp = Pregnancy complication, PTB = Preterm birth, ANC = Antenatal care, weight_gain = Weight gain during pregnancy, Age_mother = Maternal age at birth, nutria_edu = Nutritional education during pregnancy. Using the DAG model, we selected the explanatory variables to adjust the confounding pathways for the present study.

Thus, the potential risk factors of LBW and Infant mortality were selected from the DAG model. Preterm birth was defined as ‘live births before completing the gestational period of 37 weeks’, following the method given by Jana et al. [20]. The preterm birth was recoded as ‘yes’ and ‘no’. The place of delivery of the child was recoded as ‘home’, ‘private hospital’ and ‘public hospital’. The birth order of the child was recoded as ‘1’, ‘2’, and ‘3 & above’. Religion was classified as ‘Hindu’, ‘Muslim’ and ‘others’; ‘Others’ included Christian, Sikh, Buddhist/Neo-Buddhist, Jain, Jewish, Parsi/Zoroastrian, no religion and others. The place of residence was recoded as ‘rural’ and ‘urban’. National Family Health Survey-4 collected anthropometric measurements using biomarkers. Using this information, the mother who had Body Mass Index (BMI) less than 18.5 was defined as a thin mother, according to the definition of NFHS-5 [18]. The variable that a mother is thin was recoded as ‘yes’ and ‘no’. Mother’s age at birth has been categorized into ‘below 20’, ‘20-24’, ‘25-29’ and ‘30 & above’. Mother’s education was recoded as ‘illiterate or primary’, ‘secondary’ and ‘higher’. Furthermore, using the principal component analysis, the scores were generated based on the household assets such as television, bicycle, car, etc. Then the scores have been divided into two wealth quintiles. The wealth quintiles of the household were classified as ‘poor’ and ‘non-poor. The sanitation facility and sources of drinking water of the household were categorized as ‘improved’ and ‘unimproved’. Mother’s mass media exposure was recoded as ‘yes’ and ‘no’. The region of India was categorized into six; ‘North’, ‘North-East’, ‘South’, ‘Central’, ‘West’ and ‘East’.

### Statistical analysis

The study used Global Positioning System (GPS) point data to create the hotspot map of low birth weight. Local Moran’s I statistics has been used to identify the high-risk areas of low birth weight. Moran’s I provides five clustering categories, including high-high, low-low, low-high, high-low, and not significant. High-high cluster (HH) illustrates the high prevalence areas and high-risk clusters or hotspots. In contrast, low-low (LL) cluster indicates the areas of low prevalence and the low-risk clusters of incidence or cold spots [21]. Further, we estimated a two-equation model considering LBW and infant mortality separately and allowed for correlated error terms associated with each equation (cross equation correlation). For example, we observed the propensity that a mother gave birth with LBW as *y*_1_ = 1 if *y* *_1_ >  0, and *y*_1_ = 0, otherwise. We also observed the propensity that the mother found her birth (child) to death during infancy, born to her with LBW as *y*_2_ = 1 if *y* *_2_ >  0, and *y*_2_ = 0, otherwise. Below we defined the equations.

1 $$y{\ast}_1={\upalpha}_1+{\uptheta}_1+{\upvarepsilon}_1$$

where, θ_1_ = β_1_χ

2 $$y{\ast}_2={\upalpha}_2+{\uptheta}_2+{\upvarepsilon}_1$$

Where, θ_2_ = δ *y*_1_ + β_2_χ. Both equations are (standard) probit equations. $$\varepsilon ={\left({\varepsilon}_1,{\varepsilon}_2\right)}^{\prime}\sim N\left(0,\Sigma \right),\sum =\left|\begin{array}{cc}1& {\sigma}_{12}\\ {}{\sigma}_{12}& 1\end{array}\right|,{\sigma}_{12}=\rho 12$$, where ρ12 measured the endogeneity of *y*_1_ in the *y*_2_ equation. We estimated the model by exploiting cmp (conditional mixed process) command in stata [22]. Further, a multiple imputation technique has been used to impute the missing values of low birth weight in the study. Finally, model has been performed with the imputed data. We have used ArcMap version 10.3 and Stata version 14 for the analysis.

## Results

### Low birth weight, reporting system and missing data

Table 1 explains the state-wise distribution of sample children born with LBW and reporting system whether LBW outcome was reported by recalling or seeing the health card. Overall, in India, 53% of the mothers reported their babies’ birth weight by seeing health card, 36% reported by recall, and about 10% of the LBW information was observed as missing in the sample of this study. However, among the states, the missing information of LBW, means birth weight was not taken or mother did not report, was higher in Nagaland (50%) followed by Manipur (25%), Bihar (23%), Arunachal Pradesh (18%) and Meghalaya (17%), and lower percentage of missing information was observed in the South Indian states. Again, state-wise distribution of the reporting system revealed that a large proportion of mothers reported by seeing the health card which was higher in West Bengal (79%) follow by, Lakshadweep (78%), and Assam (76%). The state/union territory of Punjab and Delhi were observed to have the highest levels of LBW (about 22%), which was much higher than the national level (18.2%). However, Dadra and Nagar Haveli were observed to have 20.8% of children that are born with LBW followed by Haryana (20.5%), Madhya Pradesh (20.5%), Uttar Pradesh (20.2%) and Maharashtra (20.1%). Most of the North-Eastern states, such as Mizoram (4%), Nagaland (4.7%) and Manipur (7.2%) had the least prevalence of LBW.

**Table 1:** Distribution of sample according to their reporting of birth weight by health card, recall and the prevalence of low birth weight (LBW) and missing data across Indan states and union territories, 2019-21, *N* = 232,920

State/UTs	Total sample (N)	Reported by health card		Reported by recall		Missing		LBW	
		n	%	n	%	n	%	n	%
Jammu & Kashmir	5857	4262	72.77	981	16.75	614	10.48	560	10.69
Himachal Pradesh	2635	1714	65.05	784	29.75	137	5.20	393	15.75
Punjab	5616	2629	46.81	2690	47.90	297	5.29	1189	22.36
Chandigarh	174	95	54.60	73	41.95	6	3.45	28	16.74
Uttarakhand	3784	1593	42.10	1685	44.53	506	13.37	580	17.68
Haryana	6915	2900	41.94	3605	52.13	410	5.93	1334	20.51
Delhi	2937	1079	36.74	1677	57.10	181	6.16	608	22.09
Rajasthan	14,643	7523	51.38	6256	42.72	864	5.90	2440	17.71
Uttar Pradesh	35,766	15,660	43.78	14,710	41.13	5396	15.09	6125	20.17
Bihar	21,040	7352	34.94	8884	42.22	4804	22.83	2721	16.76
Sikkim	620	418	67.42	189	30.48	13	2.10	59	9.84
Arunachal Pradesh	5524	3173	57.44	1337	24.20	1014	18.36	479	10.63
Nagaland	3052	919	30.11	599	19.63	1534	50.26	71	4.73
Manipur	3225	1334	41.36	1086	33.67	805	24.96	174	7.23
Mizoram	2454	1243	50.65	969	39.49	242	9.86	88	4.02
Tripura	2074	1092	52.65	755	36.40	227	10.95	364	19.72
Meghalaya	6628	3120	47.07	2356	35.55	1152	17.38	642	11.73
Assam	10,645	8142	76.49	1725	16.20	778	7.31	1592	16.14
West Bengal	5618	4462	79.42	946	16.84	210	3.74	1025	18.96
Jharkhand	10,047	5666	56.39	2933	29.19	1448	14.41	1344	15.63
Odisha	8522	6109	71.69	2223	26.09	190	2.23	1596	19.16
Chhattisgarh	8514	5418	63.64	2672	31.38	424	4.98	1287	15.91
Madhya Pradesh	16,280	8253	50.69	6905	42.41	1122	6.89	3110	20.52
Gujarat	9868	5882	59.61	3647	36.96	339	3.44	1763	18.51
Dadra & Nagar Haveli	795	373	46.92	383	48.18	39	4.91	157	20.84
Maharashtra	9520	5384	56.55	3756	39.45	380	3.99	1832	20.05
Andhra Pradesh	2833	1273	44.93	1507	53.19	53	1.87	450	16.22
Karnataka	8383	5714	68.16	2468	29.44	201	2.40	1298	15.87
Goa	369	250	67.75	116	31.44	3	0.81	51	14.03
Lakshadweep	276	216	78.26	59	21.38	1	0.36	26	9.73
Kerala	2734	1975	72.24	735	26.88	24	0.88	442	16.32
Tamil Nadu	6498	4356	67.04	2098	32.29	44	0.68	1097	17
Puducherry	766	364	47.52	397	51.83	5	0.65	104	13.72
Andaman & Nicobar Island	461	197	42.73	253	54.88	11	2.39	78	17.41
Telangana	7318	3836	52.42	3341	45.65	141	1.93	995	13.87
Ladakh	529	389	73.53	101	19.09	39	7.37	56	11.61
India	2,32,920	1,24,365	53.39	84,901	36.45	23,654	10.16	38,170	18.24

Missing represents birth weight was not taken or the mother responded that she did not know. Percentage of LBW was calculated based on total reported sample

Table 2 explains the distribution of LBW, missing cases of LBW and infant mortality by background characteristics of the child, parents and households. More female children were born with LBW (16.7%) as compared to male children (14.7%). The percentage of children with LBW was found to be more among preterm babies (23.6% vs 14.5% non-preterm babies). The delivery occurring at public (16.5%) or private hospitals (17%) were found to have more LBW than the deliveries occurring at home. A high proportion of children with LBW was observed among thin (BMI < 18 kg/m^2^), below 20 years old and illiterate mothers. LBW information was observed missing to some children and the missing cases varied by different background characteristics. Out of all sample children, 17% were observed as missing the information on their birth weight status. This leads to missing the cases for the outcome variable of LBW. By background characteristics, it is revealed that 50% of the children born at home were observed to have missing information on LBW. About 20% of all illiterate mothers did not report the birth weight information. The missing cases for LBW were observed more frequent in the female births, births that were in the higher birth order, births that occurred in rural areas and births by mothers who had no exposure to mass media.

**Table 2:** Percentage distribution of low birth weight (LBW), missing LBW and infant deaths by background characteristics, 2019-21, *N* = 232,920

Control variables	Total sample (N)	LBW %(n)	Missing LBW % (n)	Infant deaths (%)
Low birth weight				
Yes	36,435			5.50
No	172,820			2.01
Sex of child				
Male	120,665	14.70 (17,736)	9.91 (11,959)	2.86
Female	112,255	16.66 (18,699)	10.43 (11,706)	2.42
Preterm birth				
Yes	29,712	23.62 (6889)	11.19 (3269)	5.76
No	203,712	14.50 (29,536)	10.01 (20,396)	2.20
Place of delivery				
Home	31,609	9.51 (3007)	50.44 (15,944)	3.24
Public hospital	150,299	16.45 (24,730)	3.76 (5651)	2.70
Private hospital	51,012	17.05 (8698)	4.06 (2070)	2.42
Birth order				
1	89,139	17.02 (15,172)	6.54 (5827)	2.68
2	76,519	15.30 (11,706)	8.41 (6437)	2.13
3 & above	67,262	14.21 (9557)	16.95 (11,401)	3.31
Mother’s age at birth				
< 20	26,445	17.89 (4731)	10.06 (2661)	3.36
20-24	99,102	16.38 (16,235)	9.25 (9170)	2.64
25-29	69,310	14.76 (10,231)	9.82 (6804)	2.36
30 & above	38,063	13.76 (5238)	13.21 (5030)	2.64
Thin mother				
Yes	48,670	14.81 (27,350)	9.95 (18,383)	2.68
No	184,670	18.83 (9085)	10.95 (5282)	2.64
Mother’s education				
Illiterate	51,210	15.70 (8042)	20.07 (10,277)	3.63
Primary	30,081	16.66 (5011)	13.93 (4191)	3.43
Secondary	119,864	15.89 (19,050)	6.96 (8339)	2.45
Higher	31,765	13.64 (4332)	2.70 (858)	1.62
Place of residence				
Rural	185,721	15.67 (29,097)	11.22 (20,843)	2.85
Urban	47,199	15.55 (7338)	5.98 (2822)	2.12
Religion				
Hindu	171,055	16.80 (28,736)	8.49 (14,527)	2.70
Muslim	33,522	14.27 (4785)	12.30 (4122)	2.55
Others	28,343	10.28 (2914)	17.70 (5016)	2.04
Wealth status				
Poor	117,869	16.07 (18,940)	15.32 (18,056)	3.31
Non-Poor	115,051	15.21 (17,495)	4.88 (5609)	2.14
Sanitation facility				
Improved	163,480	14.98 (21,614)	8.40 (12,120)	2.34
Unimproved	69,440	16.80 (11,669)	13.70 (9514)	3.26
Sauces of drinking water				
Improved	201,045	15.68 (31,531)	10.11 (20,326)	2.66
Unimproved	31,875	15.39 (4904)	10.48 (3339)	2.66
No media exposure				
Yes	121,777	15.85 (19,299)	14,60 (17,783)	3.07
No	111,143	15.42 (17,136)	5.29 (5882)	2.27
Region				
North	43,090	16.91 (7286)	7.09 (3056)	2.31
Central	60,560	17.70 (10,719)	11.47 (6945)	3.58
East	45,227	14.81 (6696)	14.71 (6652)	2.74
North-East	34,222	9.93 (3397)	16.85 (5767)	2.38
West	20,552	18.48 (3798)	3.71 (763)	2.16
South	29,269	15.51 (4539)	1.65 (482)	1.81

In the dataset, 3.4% infants died before completing their first year of age. However, this rate varied by different background characteristics. About 5.5% of infants died among children with LBW compared to 2% in children with no LBW (> 2500 g). The infant death was higher among children born at home (home delivery) (3.2%), with three or higher orders (3.3%), those born to adolescent mothers (3.4%) and those born as preterm (5.8%). Also, infant death was higher among children born to mothers belonging to the poorest households and mothers who were illiterate, who had no mass media exposure were observed.

**Fig. 3:**
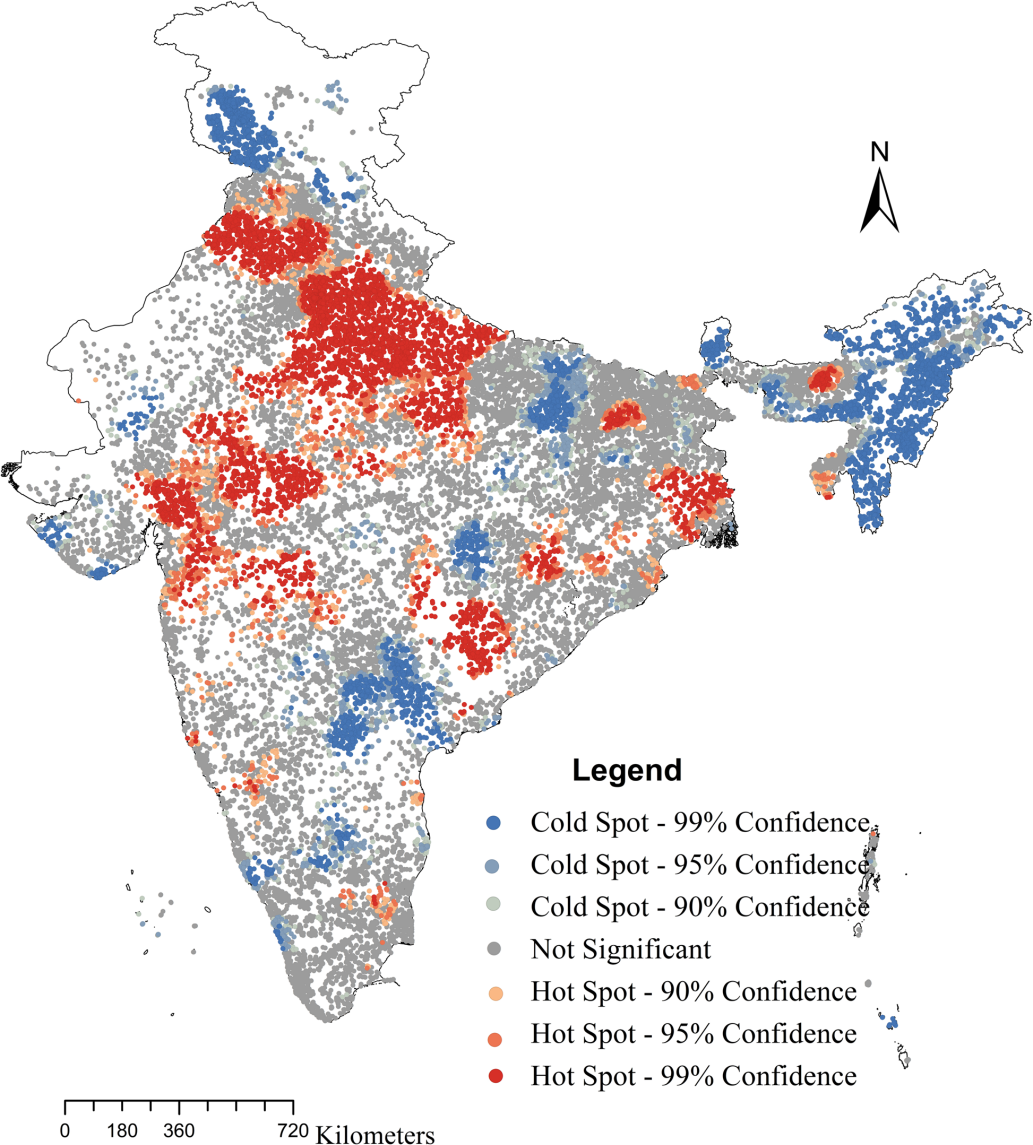
Hot spots of low birth weight in India, 2019-21

Further, we performed spatial analysis considering 30,198 clusters across all states and union territories to find out the spatial heterogeneity of LBW. Figure 3 shows the spatial distribution of the hot spots of LBW in India. The map shows that a high concentration of statistically significant hot spots were found in the Northern region, states/ union territories like Delhi, Punjab, Haryana and Uttar Pradesh. On the other hand, statistically significant cold spots were observed in the North-Eastern part of India.

### Determinants of low birth weight and infant mortality

The study exploits the conditional mixed model, which is a general framework to estimate models with various link functions. The empirical model using cmp command was estimated in other research as well [23]. We report the coefficient estimates of infant mortality (Eq. 2) (Panel A) and LBW (Eq. 1) (Panel B) in Table 3. Three models were estimated: an uncorrelated (model 1), a correlated (model 2; based on case-wise deletion; benchmark model) and a correlated model (model 3; based on imputations). The imputation is considered for the missing values against the LBW variable.

**Table 3:** Determinants of low birth weight and infant mortality in India, 2019-21, *N* = 232,920

	Coefficients (model 1) (ρ12 = 0)	Coefficients (model 2) (ρ12 <> 0)	Coefficients (model 3) (ρ12 <> 0)
Total observations	*N* = 209,255	*N* = 209,255	*N* = 232,920
Panel A (Eq. 1)			
Infant mortality (probit model)			
Low birth weight			
No®			
yes	0.45 (0.43, 0.47)***	2.02 (1.93, 2.12)***	1.82 (1.72, 1.91)***
Sex of the child			
Male®			
Female	− 0.14 (− 0.16, − 0.11)***	−0.12 (− 0.13, − 0.09)***	− 0.11 (− 0.13, − 0.09)***
Birth order			
1®			
2	0.43 (0.40, 0.45)***	0.35 (0.32, 0.37)***	0.39 (0.36, 0.41)***
3 & above	0.81 (0.75, 0.86)***	0.63 (0.58, 0.68)***	0.73 (0.68, 0.77)***
Mother’s age at birth			
Less than 20®			
20-24	−0.03 (−0.07, − 0.00)***	− 0.01 (− 0.04, 0.03)	−0.03 (− 0.06, 0.00)*
25-29	0.01 (− 0.03, 0.05)	0.05 (0.01, 0.08)**	− 0.02 (− 0.01, 0.05)
30 & above	0.08 (− 0.14, 0.04)***	0.10 (0.06, 0.14)***	0.10 (− 0.07, 0.14)***
Wealth			
Poor®			
Non-poor	−0.04 (− 0.07, − 0.01)***	−0.03 (− 0.06, − 0.01)**	−0.05 (− 0.07, − 0.03)***
Religion			
Hindu®			
Muslim	− 0.05 (− 0.08, − 0.01)***	−0.01 (− 0.04, 0.02)	−0.04 (− 0.07, 0.01)**
Others	− 0.09 (− 0.14, 0.04)***	0.02 (− 0.06, 0.03)	0.02 (− 0.06, 0.02)
Mother’s Education			
Illiterate®			
Primary	−0.03 (− 0.03, 0.04)	0.01 (− 0.02, 0.04)	0.01 (− 0.03, 0.06)
Secondary	− 0.05 (− 0.08, − 0.02)***	−0.04 (− 0.07, − 0.01)***	−0.05 (− 0.08, − 0.02)***
Higher	−0.17 (− 0.22, − 0.12)***	−0.15 (− 0.18, − 0.10)***	−0.17 (− 0.21, − 0.12)***
Place of residence			
Urban®			
Rural	0.03 (0.00, 0.06)*	0.03 (−0.00, 0.05)*	0.03 (−0.00, 0.06)*
Sanitation facility			
Unimproved®			
Improved	−0.03 (−0.06, − 0.00)**	−0.02 (− 0.05, 0.00)*	−0.02 (− 0.04, − 0.00)
Sources of drinking water			
Unimproved®			
Improved	0.00 (−0.06, 0.03)	−0.01 (− 0.04, 0.03)	−0.01 (− 0.04, 0.02)
Region			
North®			
Central	0.15 (0.12, 0.19)***	0.13 (0.10, 0.16)***	0.15 (0.12, 0.19)***
East	0.08 (0.04, 0.12)***	0.07 (0.05, 0.04)***	0.07 (0.04, 0.11)***
North-East	−0.04 (−0.05, 0.06)	−0.03 (− 0.08, 0.02)	−0.00 (− 0.04, 0.04)
West	− 0.02 (− 0.07, − 0.03)	−0.01 (− 0.06, 0.04)	−0.02 (− 0.07, 0.03)
South	− 0.05 (− 0.10, − 0.00)**	−0.03 (− 0.08, 0.01)	−0.05 (− 0.09, − 0.01)**
Panel B (Eq. 2)	*N* = 209,255		
Low birth weight (probit model)			
Preterm birth			
No®			
Yes	0.36 (0.34, 0.37)***	0.40 (0.37, 0.41)***	0.37 (0.35, 0.39)***
Place of delivery			
Home®			
Public	−0.09 (− 0.11, − 0.07)***	−0.08 (− 0.11, − 0.07)***	−0.06 (− 0.07, − 0.03)***
Private	−0.05 (− 0.08, − 0.03)***	−0.05 (− 0.08, − 0.02)***	−0.01 (− 0.04, − 0.01)
Birth order			
1®			
2	0.05 (0.04, 0.07)***	0.05 (0.03, 0.06)***	0.04 (0.02, 0.06)***
3 & above	0.15 (0.11, 0.19)***	0.13 (0.09, 0.17)***	0.09 (0.05, 0.13)***
Religion			
Hindu®			
Muslim	−0.08 (−0.09, −0.06)***	−0.07 (− 0.09, − 0.06)***	−0.07 (− 0.09, − 0.05)***
Others	−0.22 (0.25, − 0.20)***	−0.22 (− 0.23, − 0.19)***	−0.18 (− 0.20, − 0.16)***
Thin mother			
Yes®			
No	0.14 (0.12, −0.16)***	0.12 (0.10, −0.14)***	0.11 (0.09, − 0.13)***
Mother’s age at birth			
Less than 20®			
20-24	−0.05 (− 0.07, − 0.03)***	−0.05 (− 0.07, − 0.03)***	−0.05 (− 0.07, − 0.03)***
25-29	−0.09 (− 0.12, − 0.07)***	−0.09 (− 0.12, − 0.07)***	−0.09 (− 0.10, − 0.06)***
30 & above	−0.10 (− 0.13, − 0.07)***	−0.10 (− 0.12, − 0.07)***	−0.09 (− 0.11, − 0.06)***
No mass media exposure			
No®			
Yes	0.08 (0.07, 0.09)***	0.07 (0.06, 0.08)***	0.06 (0.05, 0.08)***
Correlation	–	−0.91 (−0.93, − 0.88)***	−0.90 (− 0.97, − 0.82)***

Reference categories are: panel A: infant mortality panel B: low birth weight

Model 1: The two equations are assumed independent (i.e., no correlated effects and estimated separately)

Model 2: Full model using case-wise deletion and allowing for correlation among the two error terms

Model 3: Full model using multiple imputations instead of case-wise deletion and allowing for correlation among the two error terms. Ninety-five percent confidence intervals (CIs) are given within brackets. Significance at * *p* < 0.10, ** *p* < 0.05, *** *p* ≤ 0.001;®: Reference category

Our primary focus was to estimate the effects of LBW on the probability of child deaths during infancy (0-11 months old), taking into account the frailty that the child born with LBW and death was influenced by the shared time-invariant mother specific unobserved heterogeneity, shown in panel A. Being born with LBW significantly increased the probability of death during infancy. The findings show that children born with LBW had 200% higher risk of death in the first year of life compared to children born with normal birth weight (β = 2.02, 95% CI: 1.93, 2.12). Accounting for the endogeneity of LBW (i.e., correlated errors) increased the estimated effects (marginal effects 53% in model 2 instead of 12% in uncorrelated model 1). Further, instead of case-wise deletion (model 2), the effect in a model (model 3; imputations) was estimated as less (marginal effects 48%). We consider model 3 as our benchmark (main results) model, where LBW was positively associated with the likelihood of infant mortality, and also the associated standard errors were relatively smaller compared to model 2 (correlated model with case-deletion, considering the confidence intervals). Covariates’ effects showed that female children, higher order births, births that occur in Muslim and non-poor families and to literate mothers were negatively associated with infant death. However, another set of covariates that increase the likelihood of infant deaths was: children being born to older mothers (aged 30 years and above), in rural area, and states from the Central and Eastern areas.

Results of panel B showed that preterm birth was significantly associated with the reduction in birth weight across all models. However, we explain the covariates’ effects of our benchmark model 3. The likelihood that a child will be born with LBW was 37% more in the preterm group compared to the group with ordinary births. Children being born to undernourished or thin mother, born at higher birth order, born to mothers who were uneducated or belonged to poor economic status were found to be significantly associated with LBW, while institutional birth and being born to mothers who were exposed to mass media were negatively associated with LBW.

## Discussion

Infant mortality in India has reduced from 37 per 1000 live births in 2015 to 30 per 1000 live births in 2019 at national level [24]. However, it poses a challenge to meet the SDG of reducing the infant mortality to 25 per 1000 live births by 2030. Evidence suggests that LBW is a major contributor to morbidity and infant mortality [25–28]. Children born with LBW are more common in the developing countries. A proportion ranging from 15 to 20% of all births worldwide are observed to be born with a LBW, with the highest rate in South Asian countries, i.e., 28% [29]. India alone contributes to more than 40% of the LBW newborns in developing countries and 50% in Asia [30]. Thus, this study explored the effects of LBW and the likelihood of infant mortality among children with LBW, using the latest data of national survey of NFHS 2019-21.

Earlier studies on the effects of LBW and infant mortality rarely exploits the model where it accounts for the shared mother specific frailty that influences the occurrences of LBW and influences infant death simultaneously. The current study found that half of the mothers who gave birth at their home had missing data on LBW and the multivariable estimates attenuated in the model with imputations which corroborates the previous finding that the prevalence of LBW from only the sample of measured birth weight by ignoring missing data may result in underestimation [31], and highlights the need for imputation of missing information on LBW in future studies. We estimated two equations taking into account both observed and unobserved factors. To manifest the unobserved factors that may arise failing to address all important variables in the estimation models, we allowed two correlation terms of each equation to be associated. The results of the effects of covariates were more robust in this case and provided strong evidence on the maternal, child and socioeconomic determinants of LBW and its association with infant mortality in India.

Bringing down the prevalence of LBW remains an arduous task in India. As India will not only accomplish the SDG target of reducing child mortality by preventing LBW, it may also be a push factor for human capital development [32, 33]. Although, since the 1950s, the Indian Ministry of Health has started maternal and child health care services, approximately 53% of mothers reported birth weight by health card, with significant regional disparities. This suggests the unsuccessful universal coverage of maternal and child health care services, especially institutional delivery. However, the current analysis revealed that about 18% of children were born with LBW in India. The study found an increased prevalence of LBW in Northern states such as Delhi, Punjab, Haryana and the northern part of Uttar Pradesh. Previous studies revealed that the population of Delhi and the Northern part of Uttar Pradesh are more exposed to ambient air pollution [34]. In addition, a large volume of air pollution comes from the crop residue burning that most of the farmers practice in Punjab and Haryana [35, 36]. Past evidence suggests that mothers being exposed to air pollution during pregnancy have restricted feotal growth that indicates a strong relationship between LBW and air pollution [37, 38]. This may explain having the high concentration of LBW in those regions. Furthermore, the state of Nagaland is found to have worse performance in the maternal and child health care indicators, ranking lowest among the Northeastern states [39]. Still, one out of three mothers received full antenatal care in Punjab and Haryana [18]. Although, antenatal and postnatal coverages is substantially improving in the central region of India, still a large proportion of women do not go for institutional delivery, antenatal and postnatal checkup [40, 41]. However, maternal and child health care indicators indicates the coverage of the public health programs, which are critical for LBW that is also found in our study. On the other hand, infant mortality in this study has been observed higher in the central and eastern regions which have the higher proportion of tribal population in the country, and as reported, most of the indicators related to maternal and child health are poor in these regions [42].

However, in line with our hypothesis, we noted a significant association between LBW and infant mortality. The chances of a baby dying were 53% (marginal effects in model 3) higher when the baby was born with LBW. This effect is observed about five times larger in the correlated benchmark model (model 3) compared to the effects (12%) in the uncorrelated model (model 1). A reduction of the effect is observed in the model with case-deletion (model 2) and it was 53%. The smaller marginal effect (by 5%) was observed in model 3, which may be the case that imputation for missing cases of LBW addresses the selection bias. This is shown in the covariates’ effects as well and confidence intervals (by comparing model 2 & model 3). These results evidence the importance of two steps modelling and imputation of missing observations for better conclusions.

Regarding the mechanism, LBW may cause newborn complications such as asphyxia, improper physical growth, and respiratory and metabolic dysfunction, which can increase the probability of contracting infectious diseases and malnutrition during childhood [43], which is a significant factor in reducing the survival probability of an infant [44, 45]. The factors associated with LBW in this study were premature birth, place of delivery, birth order, mother’s thinness, age at birth and exposure to mass media. Importantly, the study found that babies born before completing the 37 weeks of gestation period were more likely to have LBW—the finding supported by previous studies [46, 47]. A preterm baby gets less time in the mother’s uterus to grow and gain weight, and most of the weight of the fetus is taken during the latter part of the pregnancy [48]. In line with this, preterm birth is found to be an independent factor of LBW in our study.

Albeit the Indian government has launched Pradhan Mantri Matru Vandana Yojana (PMMVY), a conditional cash transfer scheme to improve antenatal care and institutional delivery [49], our study found that the place of delivery is a significant contributor to LBW and infant mortality. In this regard, pregnant women are eligible for the scheme if they register their pregnancy at the Anganwadi Center (AWC) within 4 months of conception; attends at least one prenatal care session; and receive iron-folic acid tablets and TT injections. Nevertheless, our study also found a lower proportion of mothers who reported birth weight by health card. It can be assumed that women who had institutional delivery have more chances of receiving health checkups and other health care services during pregnancy, which positively affects fetal growth. The WHO suggests antenatal care for pregnant women to achieve SDGs through five interventions: nutritional interventions, physical health checkups, maternal and fetal assessment, preventive measures, and health system interventions [50]. A mother needs healthy weight gain during pregnancy, which is possible through ANC visits, especially for women belonging to low-income families [51, 52]. Moreover, iron supplementation during pregnancy helps improve the mother’s nutritional status and her fetus [53]. Thus, utilizing maternal health care services increases the probability of having a healthy child. Also, it has been observed that institutional delivery raises the survival probability of newborn children with LBW as they avail the medical facilities [54, 55].

The educated and wealthy women are usually more aware of access to healthcare facilities and the risks of inadequate healthcare use than the uneducated [56]. Previous study also reported that increased awareness through media can reduce the risk of LBM and related mortality [57]. Thus, the media can potentially spread information about maternal health care that could improve health of mothers and children, especially of those with limited education [58]. Our findings also found an association between media exposure and LBW. However, parents who belong to poor economic households may not be able to afford the economic burden of hospitalization [59]. Moreover, economically wealthy families have more chances of utilizing improved healthcare facilities. Consistently, our study found a lower probability of having children with LBW and the infant deaths with the increasing mother’s education and wealth status.

Notably, the study also found that adolescent maternal age (< 20 years) is linked with an increased risk of children being born with LBW. Adolescent mothers do not have proper biological development and might not be physically or emotionally prepared to carry the fetus during pregnancy [60, 61]. A previous study based on the NFHS-4 data revealed that child marriage is associated with poor child health, i.e. LBW, due to a lack of knowledge of health and undernutrition [62]. This poor knowledge regarding maternal and child health conditions and medical care decreases the survival probability of infants [57]. In addition, a thin mother represents malnutrition, which is independently associated with LBW due to the fetus’s inadequate nutrition supply [61]. The present study also showed that babies belonging to the 1st birth order were more likely to experience LBW, which is consistent with other studies [14, 63].

This study has several strengths. Firstly, it is based on the national-level data that used validated questionnaires and methodology. Secondly, this is one among the first studies in India that address the potential risk factors of LBW and simultaneously analyze its impact on infant mortality along with other risk factors using national representative data. Lastly, this study used the imputation technique to account for the missing information.

This study had some limitations too. Firstly, the accuracy of self-reported data for the diagnosis of LBW is subject to recall and reporting bias. Secondly, as previous studies explored a strong correlation between multiple gestation and preterm birth, which has been also observed in the dataset used for the study, we could not include multiple births in the model to avoid the multicollinearity. Although maternal anemia is a good predictor of LBW, the study was not able to consider it due to the unavailability of the information.

## Conclusion

The findings of this study suggested a higher prevalence of LBW across India, which is higher in Northern India. Since LBW is shown to be associated with infant deaths in this study, it is important to prioritize the policies targeting risk factors of LBW to reduce significantly the infant mortality in India. Preterm birth is the most important predictor of LBW along with maternal factors such as delivery at home, nutritional status, age at birth and education. Measures should also be taken to improve the schemes such as iron supplementation, antenatal visit, and institutional delivery in India. The media can be used as a helpful tool for making people aware of the complications of LBW. Public-private partnerships that are recommended by the WHO in the health sector can strengthen survival outcomes of new-born babies with LBW. Through minimizing the burden of LBW, India might achieve the SDG target of reducing child mortality and malnutrition by 2030.

## Data Availability

The study uses secondary data that are available on reasonable request through https://dhsprogram.com/data/dataset_admin/.
